# Interactions of White Mugwort (*Artemisia lactiflora* Wall.) Extract with Food Ingredients during In Vitro Gastrointestinal Digestion and Their Impact on Bioaccessibility of Polyphenols in Various Model Systems

**DOI:** 10.3390/foods13182942

**Published:** 2024-09-18

**Authors:** Nacha Udomwasinakun, Shikha Saha, Ana-Isabel Mulet-Cabero, Peter J. Wilde, Tantawan Pirak

**Affiliations:** 1 Department of Product Development, Faculty of Agro-Industry, Kasetsart University, 50 Ngamwomgwan, Lat Yao, Chatuchak, Bangkok 10900, Thailand; keawnacha@gmail.com; 2 Quadram Institute Bioscience, Norwich Research Park, Norwich NR4 7UQ, UK

**Keywords:** white mugwort, *Artemisia lactiflora* Wall., polyphenol and food macronutrient interaction, in vitro bioaccessibility, antioxidant activity

## Abstract

The bioaccessibility of phytochemicals is an important factor for new functional food design. The interaction of white mugwort extract (FE) and food ingredients (coconut oil, egg white albumen, brown rice powder, inulin, and mixtures thereof) was determined after in vitro digestion to inform the development of a functional soup for an aging population. Coconut oil exerted a protective effect on polyphenols, showing the highest bioaccessibility (62.9%) and antioxidant activity after intestinal digestion (DPPH 12.38 mg GAE/g DW, FRAP 0.88 mol Fe(ll)/g DW). In contrast, egg white albumen had the most significant negative effect on the polyphenol stability, resulting in the lowest bioaccessibility (12.49%). Moreover, FE promoted the emulsion stability and delayed starch digestion by inhibiting amylase activity via non-specific polyphenol–protein interactions, resulting in a decrease in the total reducing sugars (TRS) released during digestion. It also limited the protein digestion, probably due to the complex formation of polyphenols and proteins, consequently reducing the bioaccessibility of both amino acids and polyphenols. These findings provide useful information for designing functional food products that could promote the bioaccessibility and bioactivity of natural extracts.

## 1. Introduction

*Artemisia lactiflora* Wall. (Asteraceae)—a deep green leafy plant with creamy-white flowers—is commonly called white mugwort or Jing-Ju-Chai. This plant was originally discovered in China in 1901 [1]. It has been used traditionally as a herbal medicine to treat menstrual and liver disorders. The leaves and stems are used for cooking and freshly consumed as a herbal tea owing to its bitter aromatic flavor. A few scientific studies have reported the biological activity of this plant including its antioxidant, anti-tumor, anti-inflammatory, anti-adipogenic, and anti-diabetic activity [2,3,4,5,6,7,8,9]. The bioactive compounds found in this plant are mainly phenolic acids and flavonoids, including 7-hydroxycoumarin, 7-methoxycoumarin, balanophonin, aurantiamide, aurantiamide acetate, isovitexin, kaempferol, rutin, caffeic acid ethyl ester, isoquercetin, quercetin, methyl 3, 5-di-O-caffeoyl quinate, methyl 3, 4-di-O-caffeoyl quinate, gallic acid, tannic acid, apigenin, and Catechin [4,8,10]. Generally, bioactive compounds only exert their health benefit when they are bioaccessible (released from the food matrices and available for absorption in the intestine) and bioavailable (absorbed into the body from the gut and available at the site of action) [11]. However, polyphenols undergo modifications during digestion via several factors such as pH, temperature, ionic strength, digestive enzymes, and food components, resulting in changes to their physical and functional properties [12,13]. Our previous in vitro digestion study on the bioaccessibility of white mugwort polyphenols suggested a higher bioaccessibility of the total polyphenol content (TPC) in white mugwort extract over its original powder form [14]. These compounds, mainly phenolic acids and flavonoids, are health-beneficial bioactive compounds usually incorporated as functional ingredients in foods and beverages which might interact with other food macromolecules through reversible non-covalent interactions (e.g., hydrogen, hydrophobic bonding, and van der Waals forces) and irreversible covalent bonding (e.g., through oxidation and nucleophilic addition processes) [15,16,17]. The food structure, pH, temperature, ionic strength, and relative concentration of polyphenols are factors influencing the interactions of these compounds and food macromolecules which result in both desirable and undesirable outcomes of bioaccessibility, bioavailability, and bioactivity [16].

For a better understanding, in vitro gastrointestinal digestion models are proposed to reveal these interactions because this method is an effective protocol with a lower cost, shorter turnaround time, and no ethical approval requirements. The INFOGEST static in vitro gastrointestinal model is a simple method developed by international consensus using a constant amount of ingested sample, digestive fluids, and pH for each digestion phase. In addition, the INFOGEST model has been widely used to study the interactions between plant polyphenols and food macronutrients such as starch [18,19,20], lipids [21,22,23], proteins [23,24,25,26,27], and dietary fiber [22,23,28,29]. Interestingly, both positive and negative effects of macromolecules on the bioaccessibility and bioavailability of polyphenols have been reported. Based on a literature review, there is a lack of information on the effect of the co-ingestion of white mugwort extract and food macronutrients on their bioaccessibility. Hence, the interaction between polyphenols and food macronutrients needs to be analyzed to optimize the desirable health benefits of functional foods. 

In this research, we aimed to reveal specific and mechanistic insights into the impact of mugwort polyphenol extracts (FE) on health using the INFOGEST in vitro digestion model. The suitable concentration of FE at 30 mg/mL was selected for studying the interactions with food ingredients as a model food complex for each food group including coconut oil, egg white albumen, brown rice powder, and inulin. The impact on both polyphenol bioaccessibility and macronutrient digestion was proposed to be revealed using the INFOGEST in vitro digestion model. 

## 2. Materials and Methods

### 2.1. Preparation of White Mugwort Extract and Model Food Systems

#### 2.1.1. Plant Material Preparation and Extraction

Fresh white mugwort (*Artemisia lactiflora* Wall.) (BK No. 070334) was grown and supplied by Rairuenrom Organic Farm (19°39′25.6″ N 100°09′23.9″ E) (Chiangrai, Thailand). The mature plant (40–45 days old) was harvested in October 2019 and sent to the laboratory for further extraction (50 kg). The fresh aerial parts including leaves and stems were prepared by washing and rinsing under tap water before blending at the highest speed for one minute using an electric blender (power 1000 W, FDP623WH, KENWOOD, Long Beach, CA, USA). The extraction was performed using the method described in [8]. Plant samples were extracted by blending with 95% ethanol under controlled conditions (2.5 min at 25 ± 1 °C). The mixture was separated through 0.45 µm filter paper (Whatman^®^, Piscataway, NJ, USA) using a vacuum pump. A rotary evaporator was used to remove the solvent at 45 °C. The concentrated crude extract was freeze-dried (FreeZone^®^ Plus^TM^ 6, Labconco, Kansas City, MO, USA) to obtain the dried FE extract, kept in a tightly sealed test tube and stored at −80 °C in the dark until required.

#### 2.1.2. Preparation of Model Food Systems

**Figure 1:**
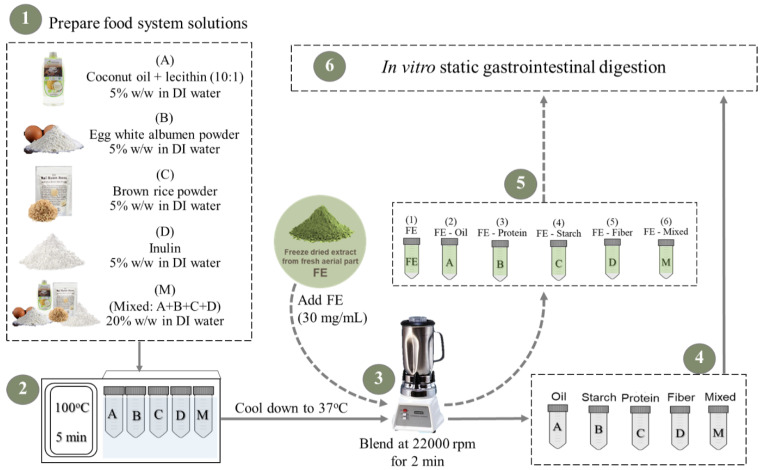
Preparation of food macronutrients in model systems.

The food ingredients used in this study included organic cold press coconut oil (Tropicana Virgin Coconut Oil, Nakhon Pathom, Thailand), egg white albumen powder (Igreca, France), brown rice powder (Organic Hom Ubon Brown Rice, Rai Ruen Rom, Cheang Rai, Thailand), and inulin powder (FS0602, CHEMIPAN, Bangkok, Thailand), and were selected to represent each macronutrient group. The five different model food/macronutrient systems, (A) oil, (B) protein, (C) starch, (D) fiber, and (M) mixed, were prepared as demonstrated in Figure 1 and as described below. 

Coconut oil was prepared as an oil in water emulsion using lecithin as an emulsifier (A). Lecithin was added to coconut oil (1:100) then stirred at 50 °C for 10 min. The solutions of egg albumen powder (B), brown rice powder (C), and inulin powder (D) were separately prepared at 5% *w*/*w* by slowly adding to deionized (DI) water while gently stirring. The system of mixed ingredients (M), including oil–lecithin, egg albumen, brown rice powder, and inulin, was mixed at the same concentrations as the individual systems and dissolved in DI water at 20% *w*/*w*. Secondly, all food macronutrient solutions (A-D and M) were heated at 100 °C for 5 min to imitate cooking conditions before cooling down to 37 °C. Each sample was homogenized by blending at 22,000 rpm for 2 min using a Waring^®^ Two-speed laboratory blender (Stamford, CT, USA). To investigate the interaction between white mugwort polyphenols and food macromolecules during in vitro digestion, known concentrations of the freeze-dried extract of white mugwort (FE) were added into each food macronutrient solution. Firstly, FE (1) was prepared by dissolving FE (30 mg/mL) in DI water before blending at 22,000 rpm for 2 min. Other model systems including FE-oil (2), FE-protein (3), FE-starch (4), FE-fiber (5), and FE-mixed (6) were prepared by adding FE (30 mg/mL) into the food solutions (A-D and M) before blending at 22,000 rpm for 2 min. 

### 2.2. Effect of Model Food Systems on Polyphenol and Antioxidant Activity of White Mugwort Extract during In Vitro Digestion

#### 2.2.1. In Vitro Gastrointestinal Digestion

**Figure 2:**
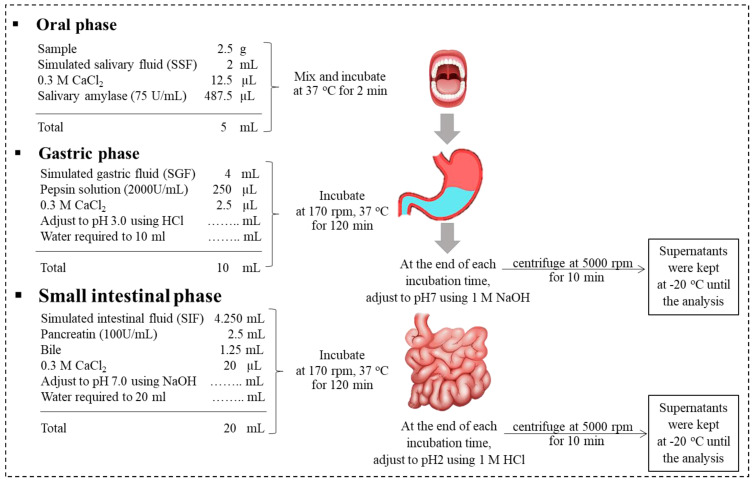
Overview of the in vitro static gastrointestinal digestion protocol (adapted [31]).

The model food systems in the presence (Figure 1: 1–6) and absence of FE (Figure 1: (A–D) and M) were subjected to standardized INFOGEST in vitro digestion [30] with minor modifications. The digestion was performed as shown in Figure 2 in separate 50 mL centrifuge tubes for oral, gastric, and gastrointestinal phases. The ingested sample (2.5 g) was mixed with simulated salivary fluid at pH 7.0 before adding salivary amylase solution (75 U/mL) (A1031-5KU, Sigma, Welwyn Garden, UK) to reach the final volume of 5 mL (Figure 2). The oral mixture was incubated for 2 min in a rotator (170 rpm, 37 °C). The simulated gastric fluid was then added to the oral mixture at pH 3.0 before adding pepsin solution (2000 U/mL) (SLCG8343, Sigma-Aldrich, UK) to reach the final volume of 10 mL. The gastric mixture was incubated for 120 min (G120) in a rotator (170 rpm, 37 °C). The simulated intestinal fluid was added to G120 at pH 7.0 before adding pancreatin solution (trypsin activity 100 U/mL) (SLBV6830, Sigma-Aldrich, UK) and bile solution (0.15 mM bile salts) (SLCD0888, Sigma-Aldrich, UK) to reach a final volume of 20 mL.

The digesta were then incubated for another 120 min (I120) in a rotator (170 rpm, 37 °C). At the end of G120 and I120, samples were adjusted to pH 7.0 and 2.0, respectively, to stop the enzyme activity and prevent further degradation of polyphenols. The collected samples were centrifuged at 5000 rpm for 10 min. The supernatants (representing the soluble, bioaccessible fraction) were collected and snap-frozen in dry ice and kept at −80 °C until the analysis of the total polyphenol content (TPC) (Section 2.2.2), antioxidant activity (Section 2.2.3), and phenolic compounds (Section 2.2.4). The experiments were performed as three independent replicates. The food systems without FE (A-D and M) were subjected to the same preparation processes and were subjected to in vitro digestion to act as a control and these baseline values were subtracted from the values for the food systems with FE ((1)–(6)) to eliminate false positive detection of reducing compounds present in the food ingredients. The bioaccessibility of TPC was calculated according to Equation (1) [31]:

(1) $$\mathrm{Bioaccessibility}\;\mathrm{of}\;\mathrm{TPC}\;(\%)=\frac{{TPC}_{soluble\;fraction}}{{TPC}_{initial\;sample}}\;\times 100$$

#### 2.2.2. Analysis of Total Polyphenol Content (TPC)

TPC was analyzed based on the Folin–Ciocalteu assay [32]. The test sample (20 μL) was mixed with Folin–Ciocalteu reagent (100 μL, 10% *v*/*v*) (Merck, Darmstadt, Germany) and Na_2_CO_3_ solution (80 μL, 7.5% *w*/*v*) (Sigma-Aldrich, Steinheim, Germany). The mixture was incubated for 30 min in a dark place. The final reaction was measured as the absorption at 760 nm using a Molecular Devices, LLC VersaMax plate reader (San Jose, CA, USA) using distilled water as a blank. The TPC value was calculated according to the standard curve of gallic acid (5–500 µg/mL, r^2^ = 0.9956) (Sigma-Aldrich, UK) and expressed as milligrams of gallic acid equivalent (GAE) per gram of sample (mg GAE/g).

#### 2.2.3. Analysis of Antioxidant Capacity

The free radical scavenging activity was analyzed based on DPPH radical assay [33]. The test sample (50 μL) was mixed with DPPH ethanolic solution (150 μL of 0.1 nM DPPH in ethanol) (Alfa Aesar, Haverhill, MA, USA). The mixture was incubated for 30 min in a dark place at room temperature. The final reaction value was determined as the absorption at 517 nm using an LLC VersaMax plate reader (USA) using distilled water as a blank. The DPPH radical scavenging activity was calculated according to the standard curve of gallic acid (2.5–100 µg/mL, r^2^ = 0.9367) (Sigma-Aldrich, UK) and expressed as milligrams of gallic acid equivalent (GAE) per gram of sample (mg GAE/g).

The ferric reducing activity was analyzed based on the FRAP assay [34]. FRAP reagent was prepared by mixing 2,4,6-Tris (2-pyridyl)-*s*-triazine (TPTZ) (40 nM in HCl) (Sigma-Aldrich, UK), FeCl_3_.6H_2_O (20 nM) (Sigma-Aldrich, UK), and acetate buffer 0.3 M, pH 3.6) (Sigma-Aldrich, UK) at a ratio of 1:1:10 *v/v* and incubating at 37 °C for 30 min prior to analysis. The test sample (20 μL) and FRAP reagent (120 μL) were mixed before being incubated for 30 min in a dark place at room temperature. The final reaction value was measured as an absorption value at 593 nm using an LLC VersaMax plate reader using distilled water as a blank. The FRAP value was calculated according to the standard curve of iron (II) sulfate heptahydrate (FeSO_4_.7H_2_O) (Sigma-Aldrich, UK) (100–1000 μmol/mL, r^2^ = 0.9903) and expressed as moles of Fe^2+^ equivalents per gram of sample (mol Fe(II)/g).

#### 2.2.4. Identification of Phenolic Compounds by HPLC-DAD

The identification of individual polyphenols was performed using liquid chromatography (LC) according to the method described by [14]. The Agilent 1100 series system instrument (Agilent Technologies 1100 Series LC, Santa Clara, CA, USA) was equipped with a quaternary pump (G1311A), automatic sampler (G1329A), and diode array detector (G1315A). The analysis was performed using a Phenomenex Luna 5u C18 (2) column (250 × 4.6 mm, 5 μm) as the chromatographic separator. The analysis temperature was set at 30 °C with the injection volume of 20 μL at a flow rate of 0.8 mL/min. The mobile phase was composed of acetic acid at 3% *v/v* (solvent A) and acetonitrile (solvent B). The gradient elution profile of solvent B was as follows: 0–8.5% (0.00–5.00 min); 8.5–2.0% (5.00–16.50 min); 2.0–18% (16.50–35.00 min); 18–20% (35.00–50.00 min); 20–30% (50.00–65.00 min); and 30–0% (65.00–70.00 min). The absorbance was recorded at 280 nm and the OpenLab CDS software (Agilent Technologies, USA) was used for result acquisition. The identification and quantification of each compound were calculated according to the standard curve of 3-CQA, 5-CQA, 3,5-diCQA, isovitexin, kaempferol 3-o-β-rutinoside, morin, rutin, quercetin, and quinic acid (r^2^ = 0.9938 to 0.9996) (Sigma-Aldrich, Gillingham, UK).

### 2.3. Effect of White Mugwort Polyphenols on the Digestion of Protein

The FE-protein and protein samples prepared as described in Figure 1 were subjected to in vitro digestion (Figure 2). The degree of proteolysis was measured using the o-phthalaldehyde (OPA) assay [35]. OPA reagent was prepared by dissolving 7.62 g of di-Sodium tetraborate decahydrate in 150 mL of DI water before adding 200 mg of SDS while stirring at 50 °C until fully dissolved. OPA (160 mg/4 mL ethanol) and DTT (176 mg) were added into the above solution. The final solution was made up to 200 mL with DI water. The reaction was performed on a 96-well plate. Each sample (24 µL) was mixed with OPA reagent (167 µL). The 96-well plate was placed and incubated for 2 min in the VersaMax^TM^ micro plate reader (USA). The final absorbance was measured at 340 nm. L-serine (0–1000 µM) was used to prepare the standard curve (r^2^ = 0.9995). Values were expressed as millimoles of L-serine equivalent per milliliter of sample (mM L-serine/mL).

### 2.4. Effect of White Mugwort Polyphenols on the Digestion of Starch

#### 2.4.1. Determination of Starch Hydrolysis

The effect of white mugwort polyphenols on starch hydrolysis was investigated by analyzing the total reducing sugar concentration released during the in vitro digestion of samples containing starch including FE-starch, FE-mixed, starch, and mixed. The PAHBAH (4-hydroxybenzoic acid hydrazide) assay was used to determine the reducing sugar concentration [36]. PAHBAH reagent was freshly prepared by dissolving PAHBAH (250 mg) in 0.5 M HCl (4.75 mL) before making up to 50 mL using 0.5 M NaOH. The reaction was prepared in micro-centrifuge tubes by mixing 20 µL of test sample with 200 µL of PAHBAH reagent. The mixtures were incubated in a water bath at 100 °C for 5 min before equilibrating for 10 min at room temperature. The final reaction was measured as an absorbance value at 405 nm using a microplate reader. Maltose (0–1000 µM) was used to prepare the standard curve (r^2^ = 0.9986). Values were expressed as millimoles of maltose equivalent per milliliter of sample (mM Maltose/mL).

#### 2.4.2. Enzymatic Inhibition

To determine any inhibitory effects of FE on enzymatic hydrolysis, brown rice powder was digested in the present of FE (0–30 mg/mL). The protocol was adjusted according to [36]. Firstly, the starch solution was prepared by dissolving brown rice powder in PBS at 30 mg/mL. The starch solution was heated at 100 °C for 5 min before cooling down to room temperature. Secondly, FE, as the potential enzyme inhibitor, was separately added into each centrifuge tube containing 10 mL starch solution to achieve final FE concentrations of 0, 5, 15, and 30 mg/mL. The solutions were then incubated at 37 °C in a rotator for 5 min. The digestion was initiated by adding 1 mL of pancreatin solution (2 U/mL) into each tube while continually incubating at 37 °C. Aliquots (100 µL) were sampled from each tube at 0, 9, 12, 15, 20, 25, and 30 min and transferred into test tubes containing 0.3 M Na_2_CO_3_ (100 µL) to stop the enzyme reaction. Samples were centrifuged at 13,000× *g* for 5 min. The supernatants were analyzed for total reducing sugars. The reaction rate (v) was calculated by dividing the concentration of total reducing sugar change (mM Maltose) by the time interval (min) [37]. 

### 2.5. Effect of White Mugwort Polyphenols on Lipid Digestion

The pH-stat auto-titration method was used to indicate the rate and extent of lipid hydrolysis during the in vitro intestinal digestion of white mugwort–lipid mixtures. Samples of FE-oil and oil prepared as described in Figure 1 were subjected to the pH-stat auto-titration method associated with the INFOGEST protocol for lipid digestion. All stimulated fluids were prepared following the INFOGEST protocol but replacing NaHCO_3_ with the same molar ratio of NaCl in order to maintain the ionic strength, as suggested by [38]. The intestinal phase digestion was performed in a titration vessel with a thermostat jacket equipped with an automatic potentiometric titrator (AT-700, KEM^TM^, Tokyo, Japan) using a titration program to monitor and maintain the pH of the mixtures at 7.0 by adding the titrant solution (NaOH 0.1 N). After 120 min, the pH-stat profile of lipolysis during the intestinal digestion was displayed as the volumes of NaOH (mL) added during digestion (min) to maintain pH 7.0. The digesta from gastric and intestinal phases were further analyzed to determine the oil droplet diameter distribution using a laser diffraction particle diameter analyzer (LS13 320, Beckman Coulter, Brea, CA, USA). The coconut oil in water emulsion and FE-oil system as well as their digesta were observed under the microscope (BX50, Olympus, Center Valley, PA, USA) to observe changes in structure such as coalescence and flocculation.

### 2.6. Statistical Analysis

The statistical analysis was performed using the SPSS Statistics for Windows, Ver. 12.0 (SPSS Inc., Chicago, IL, USA). The independent sample *t*-test was used for statistical analysis between two groups. The experiments that contain more than two groups or treatments were analyzed using one-way analysis of variance (ANOVA) and the Duncan Multiple Range Test (DMRT) at *p* ≤ 0.05 (95% significance interval). 

## 3. Results and Discussion

### 3.1. Effect of In Vitro Digestion on TPC Release and Bioaccessibility of FE in Model Food Systems

**Figure 3:**
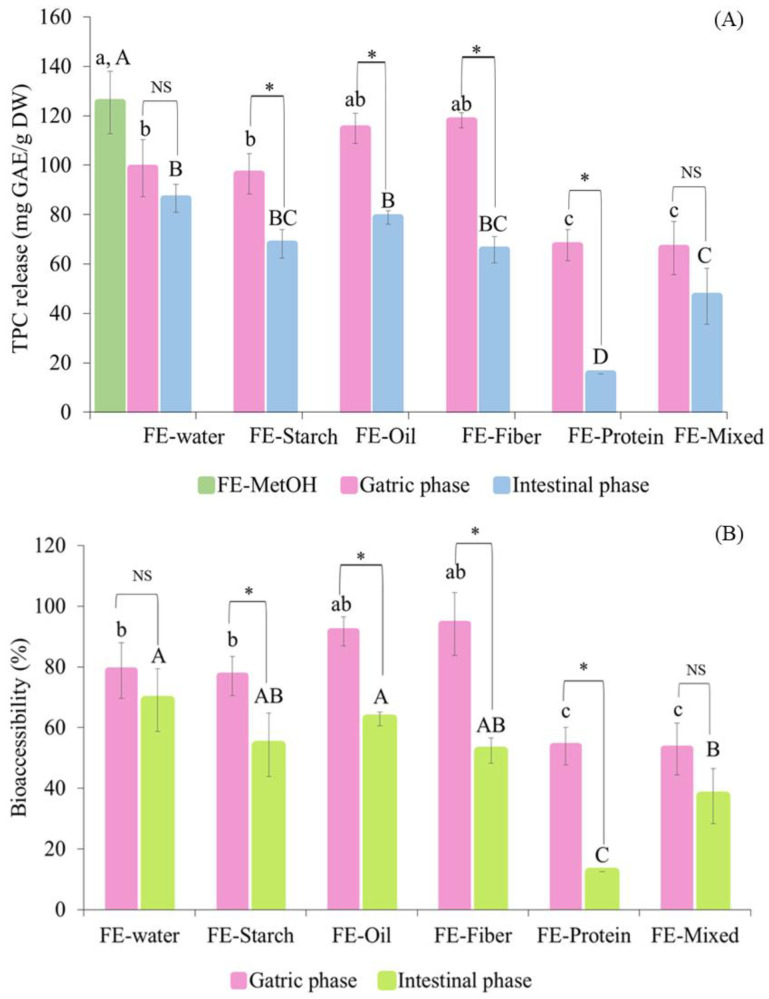
The changes in total phenolic content release (mg GAE/g DW) and bioaccessibility (%) of white mugwort extract (FE) with food systems during in vitro gastrointestinal digestion. (**A**) represents TPC release and (**B**) represents percentage of bioaccessibility. Note: Values with different letters (A–D and a–c) within the same digestion phase and (*) between digestion phases are significantly different (*p* ≤ 0.05). Differences between the two digestion phases for each food system are labeled as insignificantly different (NS) or significantly different (*) at *p* < 0.05.

To investigate the effect of food macronutrients on the bioaccessibility of TPC, the Folin–Ciocalteu assay was performed to determine the TPC release from each macronutrient sample during in vitro digestion. The presence of food macronutrients in digesta can interfere with Folin–Ciocalteu reagent due to their reducing activity. In order to obtain the accurate TPC values, the interference of food macronutrients was subtracted from the TPC values obtained for the digesta, as suggested by [39]. The digesta from gastric and intestinal phases were analyzed for the TPC and compared with FE-MetOH (FE dissolved in methanol) (125.4 ± 12.6 mg GAE/g DW) (Figure 3). 

After digestion, FE alone had a significantly lower TPC than that of FE-MetOH (*p* ≤ 0.05), suggesting the ability of the digestion process alone to reduce the TPC, owing to the changes in pH and electrolytes and possibly the presence of digestive enzymes [14,20,40,41]. The TPC release was further found to be influenced by the co-ingestion of FE with other food macronutrients. After the gastric phase, the FE-starch system (96.54 ± 8.13 mg GAE/g DW) was not significantly different to FE alone (98.82 ± 11.57 mg GAE/g DW). Although the FE-oil (114.90 ± 11.57 mg GAE/g DW) and FE-fiber systems (118.11 ± 3.03 mg GAE/g DW) did show a slight increase in TPC release compared to FE alone (98.82 ± 11.57 mg GAE/g DW), the increase was not significant (*p* > 0.05). In contrast, the co-ingestion of FE with protein significantly reduced the TPC release during gastric digestion, as found in both the FE-protein (67.61 ± 6.3 mg GAE/g DW) and FE-mixed systems (66.37 ± 10.74 mg GAE/g DW). The transition from the gastric to intestinal phase reduced the TPC release from all the sample treatments, which could be due to the instability of polyphenols under alkaline conditions, causing the degradation of these compounds [20,40,41]. After completing the digestion, the FE-protein system was found to have the lowest TPC release at 15.66 ± 0.00 mg GAE/g DW, while other treatments had slightly reduced TPC release at the end of digestion compared to that of the FE (86.58 ± 5.57 mg GAE/g DW), FE-starch (68.11 ± 5.75 mg GAE/g DW), FE-oil (78.86 ± 2.78 mg GAE/g DW), FE-fiber (65.71 ± 5.13 mg GAE/g DW), and FE-mixed systems (46.95 ± 11.35 mg GAE/g DW). These findings indicate the strong negative effect of the presence of protein on the in vitro bioaccessibility of polyphenols (as measured by the TPC analysis) compared to the effect of the other macronutrients. The co-ingestion of FE with oil, starch, and fiber had no negative impact on the bioaccessibility of the polyphenols. Interestingly, coconut oil provided a small protective effect on the bioaccessibility of the TPC in the oil in water emulsion system of the FE-oil treatment, showing a slightly greater TPC release in the gastric phase compared to that of FE, although no statistical significance was observed.

The high content of medium-chain fatty acids in coconut oil (~63%) [42] could play a role in this protective effect, since the length of the fatty acid chains was found to contribute to this effect. The formation of hydrogen bonds between the hydroxyl groups of polyphenols and the hydrophilic head of emulsifiers can lead to the higher dispersibility of medium-chain free fatty acids in the aqueous phase, thus potentially increasing the stability of polyphenols [43,44]. This idea was supported by the findings from [44], which indicated the stronger protective effect of coconut oil on the bioaccessibility and antioxidant activity of polyphenols compared to that of sunflower oil and beef tallow, representing long-chain polyunsaturated and saturated fatty acids, respectively. The emulsion of coconut oil was hydrolyzed in the small intestinal phase by the actions of pancreatic lipase facilitated by bile salts [45]. The transition from the gastric to small intestinal phase caused the destabilization of the emulsion [38]; consequently, the polyphenols were exposed to the alkaline condition, therefore leading to the reduced bioaccessibility found post-digestion of the FE-oil sample.

Similar to the coconut oil, the co-ingestion of FE with carbohydrates (FE-starch and FE-fiber) had no negative effect on the bioaccessibility of the TPC. This could be due to the known non-covalent bonding between polyphenols and carbohydrates [46] or dietary fiber contained in brown rice powder (approximately 7% dietary fiber) and inulin (approximately 96% dietary fiber) [42]. These polyphenol–dietary fiber interactions have been reported to decrease the in vitro bioaccessibility of polyphenols [23,28,29,30]. However, these polyphenol–fiber complexes can be further metabolized in the colon by the action of gut microorganisms via anaerobic fermentation [47]. The metabolized polyphenols can be absorbed in the large intestine and delivered to the liver via the hepatic portal vein prior to entering the bloodstream and being distributed to the target sites [48,49].

The limited bioaccessibility found with the co-ingestion of FE and protein was probably due to the non-specific complex formation between polyphenols and proteins, which has been widely reported [21,23,24]. The dramatic decrease in bioaccessibility found during the intestinal digestion of FE-protein (Figure 3) could be explained by the irreversible covalent interactions formed between polyphenols and proteins under alkaline conditions. The oxidation of polyphenols was induced by alkaline conditions, leading to the formation of o-quinone derivatives [50,51]. These compounds further interact with nucleophilic residues of proteins, forming protein cross-links which consequently reduce the bioaccessibility of both essential amino acids and polyphenols [52,53]. In contrast, the acidic conditions in the gastric phase favor protein hydrolysis without quinone formation; therefore, a less negative impact on bioaccessibility was observed during gastric digestion compared to in intestinal digestion [51,52,53,54]. The ingestion of the FE-mixed sample reduced the negative impact of protein on the bioaccessibility of polyphenols. This could be due to the presence of other interferences (oil, starch, and fiber) in the food matrix, possibly limiting the formation of polyphenol–protein complexes. In addition, it is important to note that the protein concentration (5% egg white albumen) was the same in both the FE-protein and FE-mixed samples.

### 3.2. Effect of In Vitro Digestion on Antioxidant Activity of FE in Food Model Systems

**Figure 4:**
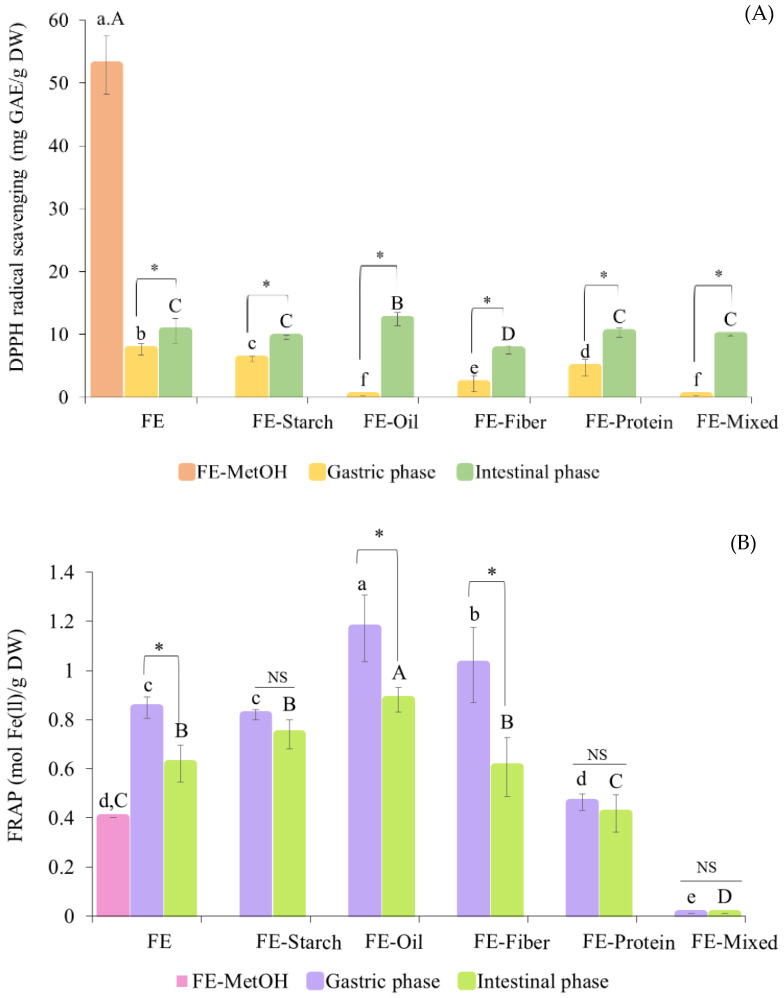
The changes in (**A**) DPPH radical scavenging (mg GAE/g DW) and (**B**) FRAP value (mol Fe(ll)/g DW) of white mugwort extract (FE) co-ingested with model food systems during in vitro gastrointestinal digestion. Note: Values with different letters (A–D and a–f) within the same digestion phase and (*) between digestion phases are significantly different (*p* ≤ 0.05). Differences between the two digestion phases for each food system are labeled as insignificantly different (NS) or significantly different (*) at *p* < 0.05).

The changes in antioxidant activity during the in vitro digestion of FE with model foods were evaluated using two different in vitro assays, the DPPH radical scavenging and FRAP assays. Initially, FE-MetOH had a DPPH radical scavenging activity of 52.87 ± 4.67 mg GAE/g DW. After digestion, all the ingested treatments had lower DPPH radical scavenging activity than FE-MetOH (*p* ≤ 0.05) (Figure 4A). After the gastric phase, the highest DPPH scavenging activity was detected in FE (7.58 ± 0.93 mg GAE/g DW) followed by FE-starch (6.08 ± 0.44 mg GAE/g DW) > FE-protein (4.72 ± 1.31 mg GAE/g DW) > FE-fiber (2.15 ± 1.26 mg GAE/g DW) > FE-oil and FE-mixed (0.02 ± 0.00 mg GAE/g DW). The transition to the intestinal phase increased the DPPH radical scavenging activity of all the ingested treatments. After intestinal digestion, the highest DPPH scavenging activity was detected in the FE-oil (12.38 ± 1.06 mg GAE/g DW) followed by FE (10.57 ± 1.97 mg GAE/g DW), FE-protein (10.25 ± 0.70 mg GAE/g DW), FE-mixed (9.80 ± 0.11 mg GAE/g DW), and FE-starch samples (9.53 ± 0.35 mg GAE/g DW).

Whilst the lowest DPPH radical scavenging activity was found in FE-fiber (7.50 ± 0.65 mg GAE/g DW), when these values were expressed as the relative DPPH radical scavenging activity percentage of the MetOH sample, they were between 14.18% and 23.42% after digestion. For the FRAP assay results, FE-MetOH displayed an activity of 0.401 ± 0.001 mol Fe (II)/g DW. After in vitro digestion, the FRAP values of the FE, FE-starch, and FE-oil samples were significantly higher than those of FE-MetOH (*p* ≤ 0.05) (Figure 4B). In contrast, the FE-mixed sample had a lower FRAP value than that of FE-MetOH. There was no statistical difference found between the FRAP values of the FE-MetOH and FE-protein samples, in either the gastric or intestinal phases (*p* > 0.05). After the gastric phase, the highest FRAP value was found in FE-oil (1.17 ± 0.14 mol Fe(II)/g DW) followed by those of the FE-fiber (1.02 ± 0.15 mol Fe(II)/g DW), FE (0.62 ± 0.08 mol Fe(II)/g DW), FE-starch (0.82 ± 0.02 mol Fe(II)/g DW), FE-protein (0.46 ± 0.03 mol Fe(II)/g DW), and FE-mixed samples (0.01 ± 0.0 mol Fe(II)/g DW), respectively. The transition to the intestinal phase reduced the FRAP values in all the treatments. After intestinal digestion, the highest FRAP value was still found in the FE-oil (0.88 ± 0.05 mol Fe(II)/g DW) followed by the FE-starch (0.74 ± 0.06 mol Fe(II)/g DW), FE (0.62 ± 0.08 mol Fe(II)/g DW), FE-fiber (0.61 ± 0.12 mol Fe(II)/g DW), FE-protein (0.42 ± 0.08 mol Fe(II)/g DW), and FE-mixed samples (0.01 ± 0.0 mol Fe(II)/g DW). When these values were expressed as a percentage relative to the FRAP value for MetOH, they varied between 0.05% and 219.9% after digestion. These findings indicated the effect of macronutrients on the antioxidant activity of polyphenols from FE. During in vitro digestion, the FRAP values were correlated to the TPC release, where the transition from the gastric to intestinal phase decreased the TPC release and FRAP values. In contrast, the DPPH radical scavenging values were increased after intestinal digestion. The different findings between the two antioxidant activity assays could be explained by the different reaction mechanisms. The FRAP assay mainly depends on electron transfer, which is the same as in the Folin–Ciocalteu assay [39]. The DPPH radical scavenging assay is based on both electron and hydrogen atom transfer [55]. The highest antioxidant activity found after the digestion of FE-oil underlined the protective effect of coconut oil on the polyphenols and antioxidant activity. Despite being in the presence of protein, the bioaccessibility of the TPC was reduced by the formation of polyphenol–protein conjugates; strong antioxidant activity has been reported resulting from polyphenol–protein reactions, since the hydroxyl groups on the polyphenols can interact with the protein [51,56]. In this study, the increased antioxidant activity after intestinal digestion was only found in the DPPH radical scavenging assay, which was consistent with the findings from [56]. In contrast, an in vivo study reported a decreased antioxidant capacity arising from the presence of polyphenol–protein interactions [51,52]. The very high relative FRAP values observed compared to the MetOH sample are thought to be in part due to the strong ability of the digestion process to extract key polyphenols, and partly due to the higher concentration of FE used for the MetOH extraction, suggesting a concentration-dependent effect on extraction efficiency, as shown in our previous paper [14].

### 3.3. Effect of Food Macronutrients on Bioaccessibility of Polyphenols from FE

**Figure 5:**
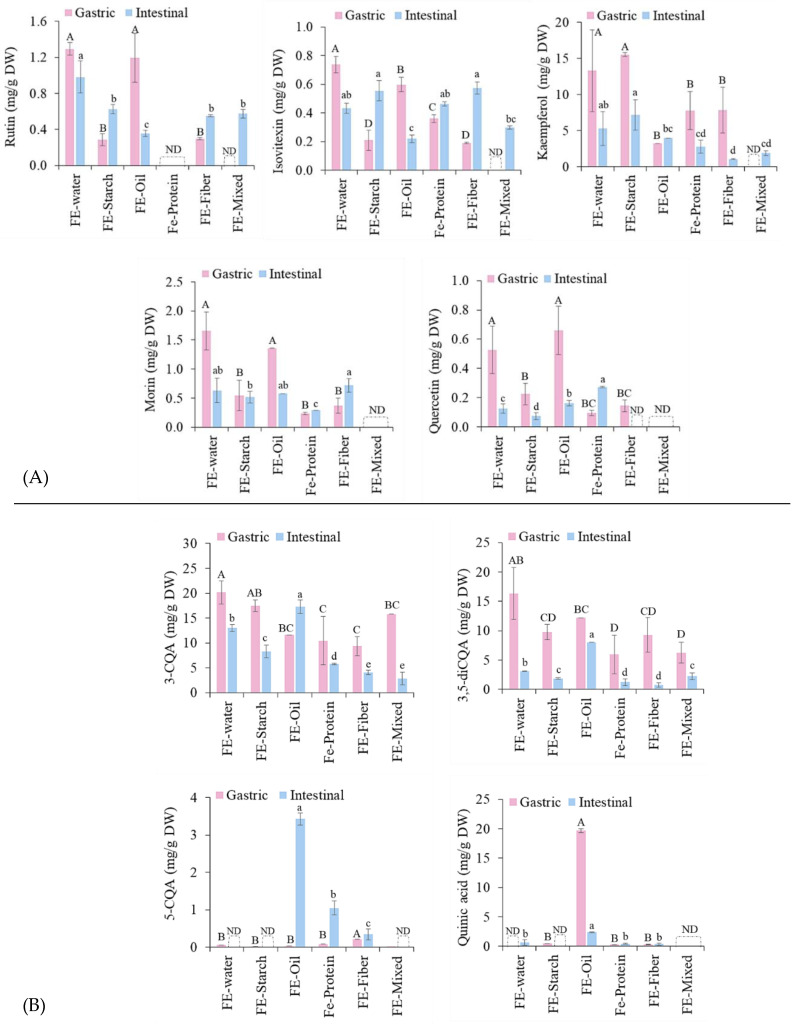
The changes in (**A**) flavonoids and (**B**) phenolic acids during in vitro digestion of FE co-ingested with food macronutrients. Values with different letters (A–D and a–e) within the same digestion phase are significantly different (*p* ≤ 0.05). ND means not detected.

According to our previous study [14] phenolic acids including 5-CQA, 3-CQA, and 3,5-diCQA as well as flavonoids including quercetin, kaemferol, morin, rutin, and Isovitexin were the major compounds in FE. In this study, during the digestion of FE with food macronutrients, the structures of these compounds could be modified which could increase or decrease their biological activity. The effect of the co-ingestion of FE with model food systems on the bioaccessibility of individual polyphenols was investigated by identifying and quantifying the polyphenols during in vitro digestion using HPLC. The changes in flavonoid and phenolic acid composition are presented in Figure 5A,B, respectively. The percentage of recovery and bioaccessibility of each compound (Table 1) were calculated relative to the polyphenol content in FE-MetOH. Most of the polyphenols showed a decreased bioaccessibility following the transition to the intestinal phase due to the alkaline conditions. These results were consistent with the TPC release (Figure 3). The in vitro digestion induced the hydrolyzation of the polyphenols and decreased their bioaccessibility. However, this hydrolysis may produce new bioactive compounds. The presence of a new compound, namely, quinic acid, might be due to the CQAs (3-CQA, 5-CQA and 3,5-diCQA) being hydrolyzed during the digestion process since CQAs are water-soluble esters of caffeic acid and quinic acid [57]. In addition, these compounds were hydrolyzed under the alkaline conditions of the intestinal phase and converted into simpler phenolics such as caffeic, ferulic, and quinic acids which can be detected in the metabolic pathway [58,59,60,61]. However, caffeic and ferulic acids were not detected in this study. Overall, the highest recovery of polyphenol compounds in the gastric phase was found in FE followed by FE-starch, FE-oil, FE-fiber, FE-protein, and FE-mixed, respectively, whereas after intestinal digestion, the highest concentrations of bioaccessible polyphenols were in FE-oil followed by FE, FE-starch, FE-protein, FE-fiber, and FE-mixed, respectively. The complexity of the various interactions between the FE and different food components resulted in the lowest bioaccessibility and antioxidant activity of the polyphenols in the FE-mixed sample. 

The highest bioaccessibility post-digestion was observed in the FE-oil sample, which further indicated the protective effect that coconut oil has on polyphenols during digestion, which was consistent with the TPC results (Figure 3). The reversal of the non-covalent interactions between polyphenols and oil taking place in the small intestinal phase may cause either an increase or decrease in these compounds. The huge increase in 5-CQA in the intestinal phase of the FE-oil sample could be due to the ability of pancreatic lipase and bile salts under alkaline conditions to hydrolyze the lipids in the emulsion [45], forming mixed micelles with the bile, which may help solubilize certain hydrophilic compounds and may therefore increase the bioaccessibility of 5-CQA in the intestinal phase. The increased quinic acid, 5-CQA, 3-CQA, 3,5-diCQA, and quercetin could contribute to the higher amount of bioaccessible TPC and greater antioxidant activity of FE-oil, as quercetin and chlorogenic acids have been suggested to possess high reducing capacity (DPPH and Folin–Ciocalteu assays) [62] and this has a high correlation with their scavenging activity [57]. Dietary fiber generally had a negative impact on polyphenol bioaccessibility in FE. The higher negative impact found in the FE-fiber over the FE-starch sample could be due to the higher dietary fiber content of inulin over brown rice powder. The binding capacity of dietary fiber for polyphenols was found to be higher than that with starch [18]. These reversible non-covalent interactions can be further metabolized by gut microorganisms before polyphenols are metabolized and reabsorbed in the large intestine [47]. Dietary fiber can also increase the viscosity of the food matrix in the small intestinal phase, which may provide health benefits by delaying glucose absorption (glycemic control) [63]. Although protein had a stronger negative impact on the recovery of polyphenols in the gastric phase compared to that of fiber, the amounts of bioaccessible polyphenols in the small intestinal phase of the FE-protein sample were higher than those of the FE-fiber sample. This could be explained by the pH changes from pH 3.0 in the gastric phase to pH 7.0 in the intestinal phase, causing the reversal of the non-covalent interactions between polyphenols and protein. This idea is supported by the study in [50] that indicated that lowering pH (pH 7.0 to 3.0) increased the ionic strength, and hence increased the binding between 5-CQA and protein via non-covalent interactions. In contrast, increasing pH (pH 4.0 to 7.0) decreased the binding sites of 5-CQA and protein, as observed in [64].

### 3.4. Effect of White Mugwort Polyphenols on Protein Digestion

**Figure 6:**
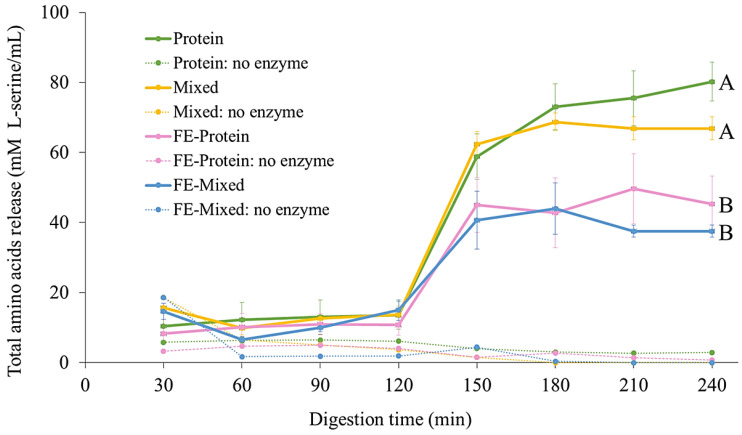
Total amino acids released during in vitro digestion of protein, mixed, FE-protein, and FE-mixed samples. Mean values ± standard deviation at the end of digestion (240 min) with different letters (A and B) are significantly different (*p* ≤ 0.05).

In addition to the strong negative impact of polyphenol–protein interactions on polyphenol bioaccessibility, antioxidant activity, and stability, these interactions may also alter protein digestion. Here, the effect of FE polyphenols on egg white albumen digestion was investigated by measuring the total amino acids (TAs) released during protein digestion of four samples, including FE-protein, FE-mixed, protein, and mixed. During the gastric phase, the TA values did not change significantly and there were no significant differences between treatments (Figure 6). This was probably because egg albumin is generally resistant to the action of pepsin in the stomach [65,66]. The TAs released then rapidly increased in the intestinal phase due to the action of pancreatic trypsin and chymotrypsin. After intestinal digestion, the highest TA values were for the protein and mixed samples in the absence of FE, whereas the FE-protein and FE-mixed samples both released significantly less TAs than the samples in the presence of FE (*p* ≤ 0.05). There was no significant difference found between the TA values of the protein and mixed samples or between the FE-protein and FE-mixed samples (*p* > 0.05). These results indicate the negative impact of FE on protein digestion. This could be due to the complex formation between polyphenols and protein, which has been widely reported [23,24,27], that restricts the access of the enzymes to the protein substrate. Furthermore, the formation of o-quinone generated from the oxidation of polyphenols under alkaline conditions in the small intestine may also interact with the nucleophilic residues of the protein, forming protein cross-links, consequently reducing the bioaccessibility of both essential amino acids and polyphenols [50,51]. These results support the reduced bioacessibility found with the co-ingestion of FE and protein.

### 3.5. Effect of White Mugwort Extract Polyphenols on Carbohydrate Digestion

#### 3.5.1. Determination of Total Reducing Sugars (TRS)

**Figure 7:**
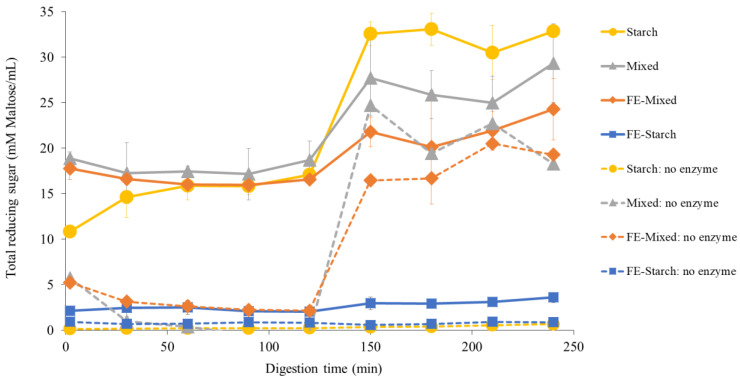
Total reducing sugars released during in vitro digestion of starch, FE-starch, and FE-mixed samples. Gastric phase was 2–120 min and intestinal phase was 120–240 min.

The model food system samples including FE-starch, FE-mixed, and starch were analyzed for TRS released during in vitro digestion using the PAHBAH assay (Figure 7). 

Each sample which contained starch was subjected to in vitro digestion in the presence and absence of α-amylase and pancreatic amylase and in the presence and absence of FE. Without digestive enzymes, as expected, the TRS released from the starch and FE-starch samples were not increased during digestion. However, there was an increase in the TRS in the oral and intestinal phases of the mixed and FE-mixed treatments without digestive enzymes. This could be due to the presence of an alkali-labile free sugar fraction in inulin powder. Therefore, the TRS was detected under the alkaline conditions (pH 7.0) of the mixed and FE-mixed (no enzyme) samples [40,62,67]. In the presence of digestive enzymes, the highest TRS was found in the starch followed by mixed, FE-mixed, and FE-starch samples (Figure 7). The transition from the gastric to the small intestinal phase showed a sudden increase in the TRS. This was due to the action of pancreatic amylase which hydrolyses starch [68]. The co-ingestion of FE with starch drastically lowered the TRS released during digestion compared to that of the sample in the absence of FE (starch), indicating a strong inhibition of starch-digesting enzymes. The presence of other macronutrients in the FE-mixed sample reduced these inhibitory effects. These findings indicate the inhibitory effect of polyphenols from FE on starch digestion, which was further investigated using enzyme inhibition assays.

#### 3.5.2. Enzymatic Inhibition

**Figure 8:**
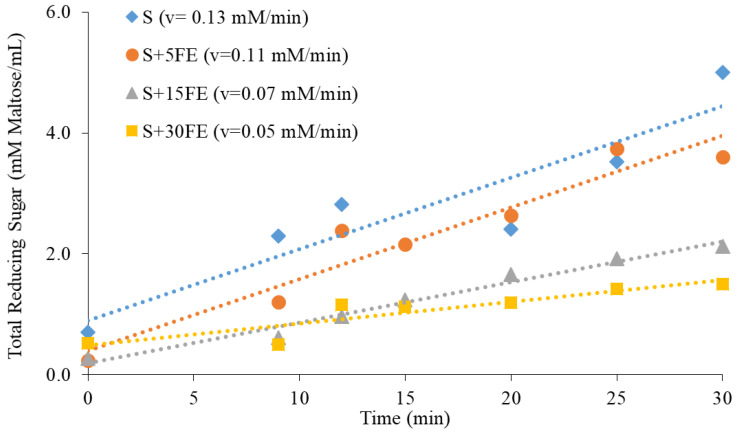
The effect of white mugwort extract polyphenols on starch digestion.

In order to study the inhibitory effect of FE polyphenols on the activity of pancreatic amylase further, brown rice powder was co-ingested with different concentrations of FE 0–30 mg/mL). The PAHBAH assay was used to determine the hydrolysis rates of starch by pancreatic amylase (2 U/mL) by measuring the TRS released during starch amylolysis. The reaction rate of starch amylolysis is indicated by the change in the concentration of the TRS over time (v = mM Maltose/min) [37]. The reaction rates (Figure 8) were decreased with the increase in the FE concentration, indicating the ability of polyphenols from FE to slow starch hydrolysis. This could be due to the the inhibitory effect on polyphenols on amylase activity though the non-covalent interaction of the polypenols with the enzyme restricting access to the active site. This reaction occurs between the hydroxyl groups of phenolic and polar groups (e.g., -OH, -SH, -NH) of proteins such as amylase [51].

This idea was supported by several studies. For example, Ref. [69] indicated that the interaction between digestive enzymes and either pure polyphenols or crude plant extracts caused changes in the secondary structure of proteins (α-helix and β-sheet), resulting in protein unfolding and a subsequent decrease in enzymatic activity. This enzymatic inhibitory effect of polyphenols is desirable for the nutritional improvement of carbohydrate-rich diets, mainly by reducing the glycemic response [70]. The inhibitory effect of white mugwort extract on digestive enzymes such as D-amylase and D-glucosidase was also reported in [7]. These findings indicated the health benefits of white mugwort extract through reducing the risk of type 2 diabetes, owing to its ability to delay or slow starch hydrolysis, glucose absorption, and further improve glycemic control [46,71,72].

### 3.6. Effect of White Mugwort Polyphenols on Oil Digestion

**Figure 9:**
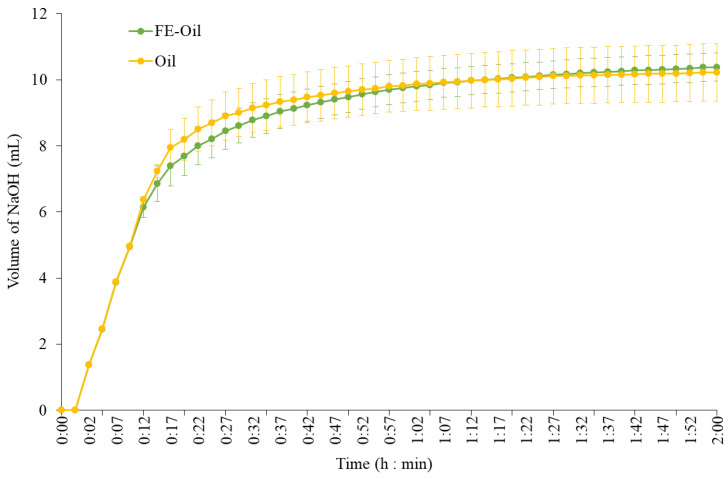
pH-stat profiles measured during the intestinal phase of in vitro digestion for the coconut oil emulsion with and without white mugwort extract.

During lipid digestion, digestive lipases hydrolyze triglyceride molecules, releasing free fatty acids and monoglycerides from the glycerol backbone, resulting in a decrease in the pH of the digesta [73]. Therefore, the effect of FE polyphenols on coconut oil emulsion digestion was investigated by monitoring the free fatty acids released during lipolysis by pancreatic lipase using the pH-stat titration method to maintain the pH during intestinal digestion (Figure 9). At 12 to 52 min, FE-oil required a slightly lower volume of NaOH compared to the oil sample, indicating that coconut oil lipolysis had been delayed when FE was present. However, the pH-stat profiles of both samples were not significantly different (*p* > 0.05). The final levels of NaOH added to oil and FE-oil were 10.22 and 10.38 mL, respectively. These results indicated that FE had no significant effect on the lipase activity or free fatty acids released during coconut oil emulsion digestion.

**Figure 10:**
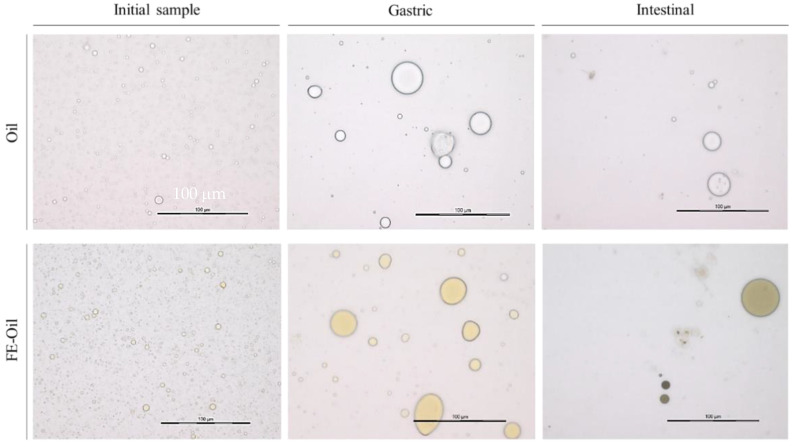
The changes in emulsion droplet of coconut oil emulsion with and without white mugwort extract during in vitro digestion.

The physicochemical properties of emulsions such as the droplet diameter and specific surface area (SSA) can affect the lipolysis rates of lipase and alter fat absorption. The reduction in the surface area-to-volume ratio of oil contributes to a lower lipid digestion rate, as it effectively determines the availability of the interface for adsorption by digestive lipases to the surface of oil droplets. Droplets of the emulsions of the oil-FE and oil samples were observed using light microscopy (Figure 10). The presence of FE slightly increased the emulsion droplet diameter distribution of coconut oil in the o/w system. The increase in the droplet diameter results in a reduction in the SSA. Table 2 shows that the FE-oil sample had higher median and average droplet diameters than those of the oil sample and lower SSA values compared to the oil sample.

However, a statistically significant difference was only found in the median droplet diameters between the oil and FE-oil samples (*p* ≤ 0.05). Reference [74] also reported an increased emulsion droplet diameter and decreased SSA when polyphenols from black tea were added to an olive oil emulsion. These results indicated an effect of polyphenols on the physicochemical properties of emulsions, possibly caused by the interaction between polyphenols and emulsifiers. These compounds interact through the hydrogen bonds between the hydroxyl groups of polyphenols and the hydrophilic head of emulsifiers, making them less efficient emulsifiers and thus increased the emulsion droplet diameter and decreased SSA [43,44]. These interactions appear to be desirable due to the positive effect on the bioaccessibility of polyphenols (Figure 3). In addition, these interactions could further reduce lipid digestion and absorption rates [11].

Initially, the average droplet diameter in the oil sample was found to be 2.01 µm. The gastric phase increased the average droplet diameter in the oil sample to 42.0 µm (Table 2). These large particles were rapidly broken down in the small intestine, leading to the presence of a smaller average droplet diameter at 10.9 µm. The rapid changes in the droplet diameter distribution in the small intestine could be explained by several modifications occurring in the intestinal phase, mainly the action of bile salts to break up larger fat aggregates into small droplets and increase the SSA, which makes it easier for the pancreatic lipase to access and hydrolyze lipids, leading to the presence of smaller droplets [75,76]. Some larger droplets found in the intestinal phase could have been due to the aggregation or coalescence of lipid droplets or the formation of mixed micelles [77]. In comparison, the SSA and mean droplet diameter in the oil and FE-oil samples showed no significant difference in the gastric phase (*p* > 0.05), whereas the intestinal phase increased the SSA in the oil sample ((7.0 ± 1.3)10^6^ cm^2/^g), showing a significantly higher SSA value than in the FE-oil sample ((6.9 ± 0.9)10^5^ cm^2/^g) (*p* ≤ 0.05). The median droplet diameter was decreased in the intestinal phase of both samples. The inversion between the SSA and particle diameter is expected to be found because the SSA of an emulsion is reduced when small droplets coalescence to form larger droplets [72]. After digestion, the median droplet diameter in the FE-oil sample (20.4 ± 2.1 µm) was significantly higher than that in the oil sample (10.9 ± 2.8 µm), implying that the FE increased the stability of the coconut oil emulsion. These findings can be used to explain the protective effect of coconut oil emulsion, which could increase the bioaccessibility of polyphenols in FE-oil samples.

## 4. Conclusions

This work has revealed the impact of interactions between polyphenols in mugwort extract and food macronutrients in model food systems during in vitro gastrointestinal digestion. A coconut oil emulsion appeared to exert a protective effect on the polyphenols, resulting in the highest polyphenol bioaccessibility and antioxidant activity after intestinal digestion. In contrast, the presence of protein (egg white albumen) imparted the strongest negative effect on the polyphenol bioaccessibility. The mixture comprising all the macronutrients also had a strong negative impact on the polyphenol bioaccessibility, probably due to the complex interactions between the polyphenols and the various macronutrients. Carbohydrates (starch and fiber) also showed a negative effect on the antioxidant activity and polyphenol bioaccessibility. 

The interactions between polyphenols and food macronutrients also affected macronutrient digestion. Whilst FE did not affect lipid digestion per se, FE did appear to increase the stability of the coconut oil emulsion during digestion, which may have contributed to the protective effect of coconut oil on the polyphenol bioaccessibility and antioxidant activity. However, FE also delayed starch digestion by inhibiting amylase activity via polyphenol–protein interactions. FE was also found to limit protein digestion due to the aggregation between polyphenols and proteins, consequently reducing the bioaccessibility of both the amino acids and polyphenols. These findings provide useful information for formulating foods that can promote the bioaccessibility and bioactivity of white mugwort polyphenols.

## Figures and Tables

**Table 1:** Changes in phenolic compounds of white mugwort extract co-ingested with food systems during in vitro digestion.

Compounds	FE-MetOH (mg/g)	%	FE	FE-Starch	FE-Oil	FE-Fiber	FE-Protein	FE-Mixed
5-CQA	0.3 ± 0	R_G_	150	135	28	177	154	3.4
		BA_I_	ND.	ND.	1319	130	402	ND.
3-CQA	22 ± 2	R_G_	90.6	79	53	42	47	71
		BA_I_	58.4	37	78	18	26	13
Rutin	1.8 ± 0	R_G_	73.9	16	68	17	ND.	ND.
		BA_I_	56.2	36	20	32	ND.	33
Isovitexin	0.9 ± 0	R_G_	80.1	23	65	21	39	ND.
		BA_I_	46.9	60	24	62	50	32
Kaempferol	5.3 ± 1	R_G_	249	291	61	147	145	ND.
		BA_I_	99	134	74	20	52	35
3,5-diCQA	20 ± 6	R_G_	82.2	49	61	47	30	31
		BA_I_	15.7	9.3	40	3.7	6.2	11
Morin	2.2 ± 0	R_G_	76.4	25	63	17	11	ND.
		BA_I_	29.1	24	27	33	13	ND.
Quercetin	0.7 ± 0	R_G_	53.1	34	100	22	15	ND.
		BA_I_	12.7	11	25	ND.	42	ND.

R_G_ = Recovery (%) of each compound after gastric phase. BA_I_ = Bioaccessibility (%) of each compound after intestinal phase. R_G_ and BA_I_ were calculated relative to the polyphenol content in FE-MetOH. ND. = Nondetectable.

**Table 2:** The changes in specific surface area and droplet diameter of coconut oil emulsion with and without white mugwort extract during in vitro digestion.

Droplet Distribution	Initial Sample	Gastric		Intestinal	
Specific surface area (cm^2^/g)		Oil	(5.3 ± 0.9) × 10^6^ ^A,ns^	(2.1 ± 0.4) × 10^5 B,ns^	(7.0 ± 1.3) × 10^6 A,a^
		FE-Oil	(4.3 ± 1.6) × 10^6 A,ns^	(6.2 ± 1.5) × 10^5 B,ns^	(6.9 ± 0.9) × 10^5 B,b^
Median droplet diameter (µm)		Oil	1.62 ± 0.12 ^B,b^	43.8 ± 6.6 ^A,ns^	17.6 ± 2.9 ^B,ns^
		FE-Oil	40.3 ± 11.1 ^C,a^	40.3 ± 11.1 ^A,ns^	37.9 ± 17.2 ^B,ns^
Average droplet diameter (µm)		Oil	2.01 ± 0.27 ^C,ns^	42.0 ± 5.5 ^A,ns^	10.9 ± 2.8 ^B,b^
		FE-Oil	2.60 ± 0.67 ^B,ns^	39.0 ± 10.4 ^A,ns^	20.4 ± 2.1 ^A,a^

Mean values ± standard deviation with different letters between digestion phase (A–C) and between samples (a and b) are significantly different (*p* ≤ 0.05). *p*-value higher than 0.05 is represented by ns (non-significant difference).

## Data Availability

The original contributions presented in the study are included in the article, further inquiries can be directed to the corresponding authors.
